# The Maximal Intensity Period: Rationalising its Use in Team Sports Practice

**DOI:** 10.1186/s40798-022-00519-7

**Published:** 2022-10-12

**Authors:** Dan Weaving, Damien Young, Andrea Riboli, Ben Jones, Giuseppe Coratella

**Affiliations:** 1 Carnegie Applied Rugby Research (CARR) Centre, Carnegie School of Sport, Leeds Beckett University, Leeds, West Yorkshire UK; 2 Leeds Rhinos Rugby League Club, Leeds, West Yorkshire UK; 3 Technology University of the Shannon, Midlands Midwest. Thurles Campus, Thurles, Tipperary, E41 PC92 Ireland; 4 Department of Biomedical Sciences for Health, Università degli Studi di Milano, via Giuseppe, 20133 Colombo 71, Milano Italy; 5 England Performance Unit, The Rugby Football League, Leeds, UK; 6 School of Science and Technology, University of New England, Armidale, Australia; 7 Division of Exercise Science and Sports Medicine, Department of Human Biology, Faculty of Health Sciences, The University of Cape Town and the Sports Science Institute of South Africa, Cape Town, South Africa

**Keywords:** Team sports, Soccer, Global positioning system, Rolling average, Performance, Football, Training, Small-sided games, Time–motion analysis

## Abstract

Quantifying the highest intensity of competition (the maximal intensity period [MIP]) for varying durations in team sports has been used to identify training targets to inform the preparation of players. However, its usefulness has recently been questioned since it may still underestimate the training intensity required to produce specific physiological adaptations. Within this conceptual review, we aimed to: (i) describe the methods used to determine the MIP; (ii) compare the data obtained using MIP or whole-match analysis, considering the influence of different contextual factors; (iii) rationalise the use of the MIP in team sports practice and (iv) provide limitations and future directions in the area. Different methods are used to determine the MIP, with MIP values far greater than those derived from averaging across the whole match, although they could be affected by contextual factors that should be considered in practice. Additionally, while the MIP might be utilised during sport-specific drills, it is inappropriate to inform the intensity of interval-based, repeated sprint and linear speed training modes. Lastly, MIP does not consider any variable of internal load, a major limitation when informing training practice. In conclusion, practitioners should be aware of the potential use or misuse of the MIP.

**Key Points**

- Determining the maximal intensity period can inform the training of the most intense demands in team sports matches
- Maximal intensity period can be used as a benchmark for sport-specific drills such as small-sided games
- Maximal intensity period underestimates the actual player’s physical capacities and should not be used as a benchmark in running-based exercises

## Background

Measuring the time–motion characteristics of male and female team sports including rugby union [1, 2], rugby league [3], soccer [4], Australian football (AFL) [5, 6], Gaelic football [7] and hurling [8–11] has been of interest to researchers and practitioners for decades. Such data can be generated by video, semi-automated camera systems, global positioning systems (GPS), global navigation satellite systems (e.g. GLONASS, Galileo and BeiDou) [12] and tri-axial accelerometer-based methods [8, 9, 13]. Specifically, time–motion analysis is used to profile the external intensity, frequency and duration of the activities completed by players. Nevertheless, given the intermittent nature of team sports, the commonly used time–motion variables include total distance discretised into various speed thresholds (e.g. high-speed running and sprinting), average speed and changes in speed (i.e. accelerations and decelerations) [14, 15]. Collectively, these data are used to determine the performance model and to inform the training process by allowing comparisons between match play and training prescription [1–5, 7–9, 16].

**Fig. 1:**
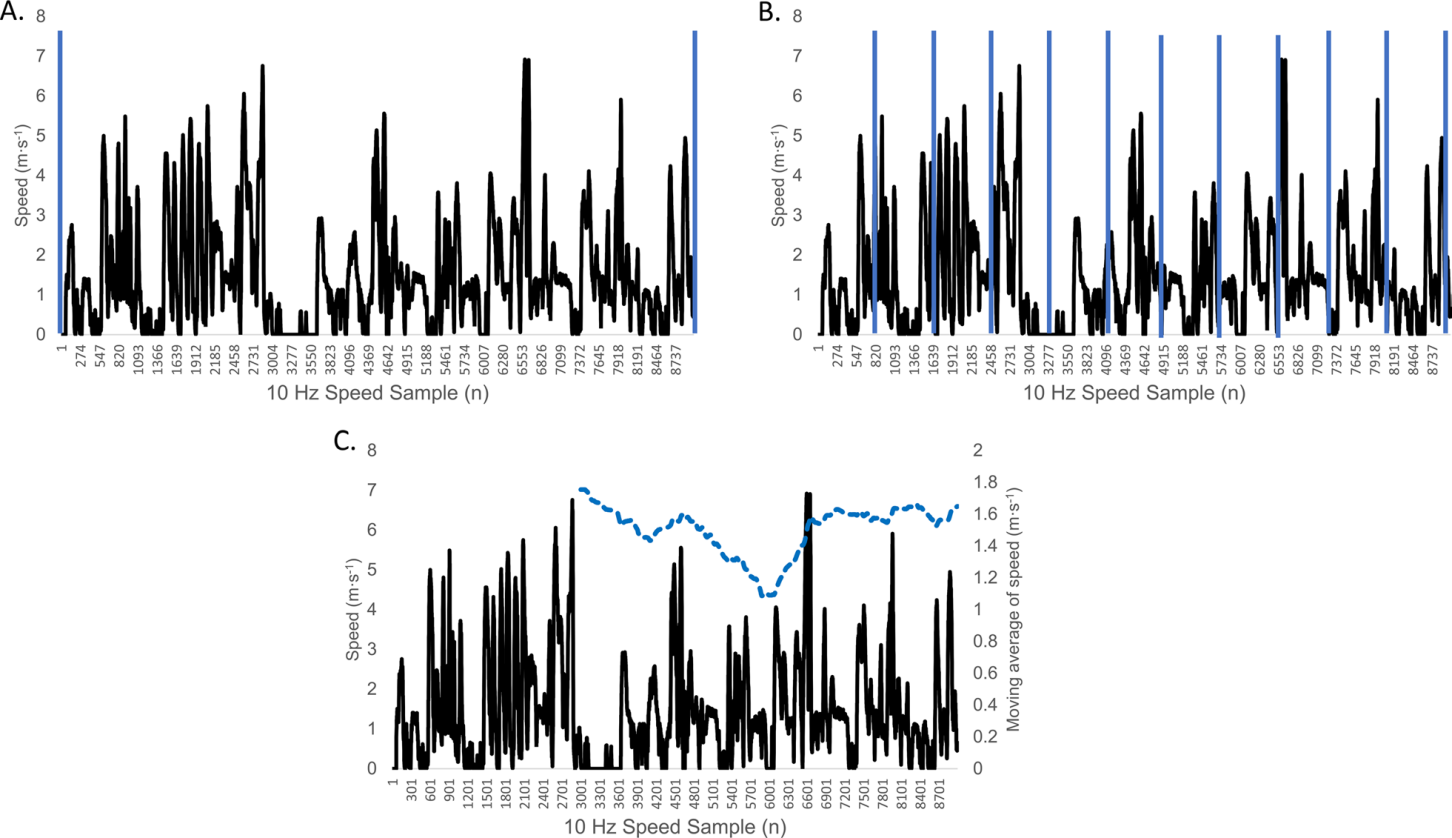
Example of global positioning system data and common methods of analysis: whole period (**A)**, segmental (**B**) and moving average (**C**)

An important consideration for the time–motion analysis is how metrics are represented over time to reflect the intensity of a match, i.e. the rate of activity completed by players [17]. Traditionally, metrics have been represented by segmenting the data over predefined durations, often the full match, per quarter/half or smaller predefined durations (e.g. 0 to 5, 5 to 10 min) (Fig. 1-A and 1-B) [5, 7, 8]. However, by averaging the match demands across these durations, there is an underrepresentation of the highest intensity experienced during match play for any given duration [5, 7, 8]. To overcome this limitation, a moving average approach was proposed to allow researchers and practitioners to identify the maximal intensity for different durations for a number of variables (Fig. 1-C) [1, 18–20]. Such an approach consists of taking the data sampling at a given frequency (e.g. speed sampling at 10 Hz), applying a moving mean for a given duration (e.g. 1 min = mean every 600 samples), and then taking the maximum moving average value. This permits the identification of the highest intensity for a given duration of time, which is often referred to as the ‘peak demand’ ‘peak locomotor demand’, ‘peak characteristic’, ‘duration-specific locomotor demand’, ‘maximal intensity period’ or ‘worst case scenario’ [15, 18, 21, 22]. From now on, we will refer to this as the maximal intensity period (MIP).

The aim of the MIP is to appropriately represent the maximal intensity experienced by the athlete, to avoid an underrepresentation of the match play intensity when considering whole- or half-match average data only [15, 18, 23]. These MIPs (calculated over varying durations) have been suggested to provide ‘benchmarks’ to evaluate training prescription and could be useful to replicate the match intensity during specific training drills, such as the small-sided games (SSGs) [20]. However, a recent paper questioned the appropriateness of the MIP approach to define the training content [21]. Conceptually, the authors questioned the sole use of time–motion (or external intensity and volume) data to inform the training prescription since this would provide limited insight into the psycho-physiological responses to training [21]. Consequently, measuring the internal response to such data was also necessary to understand the likely training-induced adaptations [21]. The authors’ concerns were also based on the high between-match variability of such measures and a number of contextual factors (﻿e.g. when MIP occurred, starter or non-starter player, total game time of the player, the volume of activity performed directly before the MIP) that could influence the MIP [21].  Although, another study reported lower variability in the 1-min MIP for the high-intensity activities and reported that the 1-min MIP was only partially affected by further match-related factors (e.g. formation, ball-in-play, and ball-out-of-play) [23]. However, should coaches focus only on the mean demands over the game, the MIP may be overlooked and the possible benefits deriving from the use of the MIP should still be considered.

While these match-related factors are relevant, previous research has not clarified or rationalised which training prescription variables or modes the MIP data could be reasonably and logically applied across in the overall training process. Therefore, within the present conceptual review, we aimed to firstly describe how MIP can be determined; secondly, compare between whole-game average and MIP methods within a number of team sports; thirdly, rationalise when MIP  could or should not be used in the training practice, highlighting both the strengths and weaknesses when referring to MIP as a training prescription benchmark; lastly, address future research to clarify the unsolved points.

### MIP in Team Sports

#### The Determination of the MIP

In team sports, the most commonly used GPS units collect data at 10 Hz; therefore, data can be extracted post-match at 0.1-s intervals. Once downloaded, the traditional approach is to analyse the time–motion data across the whole game, per half or per quarter (Fig. 1-A). However, data can also be divided across phases of play (e.g. attack and defence), by ball-in-play periods and using a moving average method across different epoch durations (e.g. 1–10 min) (Fig. 1-B) [18, 20, 23]. For example, for the 1-min epoch duration, the moving average method is based on moving 1-min epochs starting at each 0.1-s intervals. Logically, as the duration of the moving average increases, the MIP value decreases [18, 19, 24]. To account for the MIP across all chosen durations, a power-law relationship can be also determined [25]. This involves conducting a linear regression of the log–log relationship between the time interval (s) on the x- and the distance covered (m) on the y-axis to derive the intercept and slope (Fig. 1-C) [25]. As such, the MIP could be determined from continuous rather than discrete time windows, providing a deeper overview of the time–motion characteristics across different MIP durations.

#### Whole Match vs. MIP

**Fig. 2:**
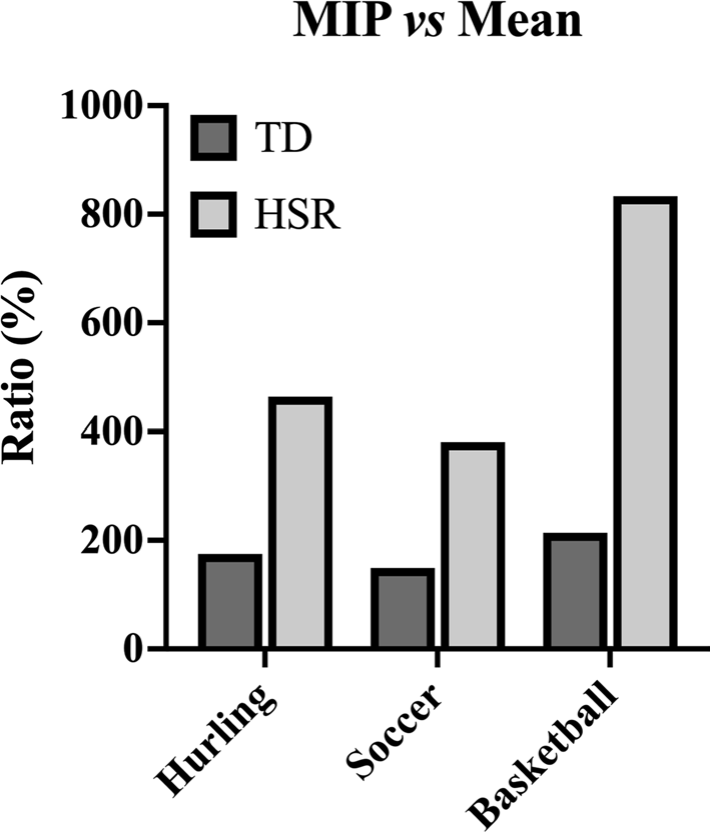
The ratio (%) between the mean and MIP per min for the total relative distance (black bars) and high-speed running distance (white bars) in hurling (4.7–6.1 m⋅s^−1^), soccer (4.2–5.6 m⋅s^−1^) and basketball (> 5 m⋅s^−1^). The data for the direct comparison are taken from hurling [18], soccer [23] and basketball [26]. MIP: maximum intensity period. HSR: high-speed running

The need to consider the MIP data can be explored by examining their difference with the mean whole-match analysis by comparing relative distances within different speed zones. Indeed, data from a direct comparison within some team sports highlight that the MIP over a 1-min period appears far greater than the mean values from the whole match for total distance (hurling: 175%; soccer: 149%; and basketball: 214%), high-speed distance (hurling [4.7–6.1 m⋅s^−1^]: 464%; soccer [4.2–5.6 m⋅s^−1^]: 381%; and basketball [> 5.0 m⋅s^−1^]: 833%) (Fig. 2), very high speed distance (soccer [5.6–6.7 m⋅s^−1^]: 450%) and sprint distance (hurling [> 6.1 m⋅s^−1^]: 600%, and soccer [> 6.7 m⋅s^−1^]: 724%) [15, 18, 26]. Given the importance and the relevance of the question, a systematic review in soccer detailed how the MIP relates to whole match play analysis when accounting for time epoch and position [27]. Although further direct comparisons between the MIP and mean within other team sports are not available, the data compared between different studies with comparable elite senior players and the same speed zone thresholds showed that the MIP is far greater than the data presented as the mean only. For example, total distance in rugby union (MIP =  ~ 171 m·min^−1^ vs mean =  ~ 68 m·min^−1^) [20, 28], rugby league (MIP =  ~ 165 m·min^−1^ vs mean =  ~ 90 m·min^−1^) [29, 30], Australian football (MIP =  ~ 210 m·min^−1^ vs mean =  ~ 131 m·min^−1^) [24, 31] and Gaelic football (MIP ~ 225 m·min^−1^ vs mean =  ~ 131 m·min^−1^) [29, 30] is 251%, 170%, 160% and 172% respectively greater when determined using MIP vs mean values. Moreover, the ratio between the MIP vs mean is 900% in rugby union (> 5.0 m⋅s^−1^) (MIP =  ~ 54 m·min^−1^ vs mean ~ 6 m·min^−1^) [1, 28] and 287% in Gaelic football (> 4.7 m⋅s^−1^) (MIP =  ~ 43 m·min^−1^ vs mean =  ~ 15 m·min^−1^) [7, 32] for high-speed running. Further comparisons are not possible as they would imply extracting data from different populations and speed zone thresholds within each team sport. Notwithstanding, the message is clear: due to the inclusion of periods of inactivity, the mean values largely underestimate the intensity of the match, and the magnitude of underestimation increases with higher speed thresholds and shorter time durations.

However, the MIP refers to very short arbitrary durations (e.g. 1 to 10 min) and therefore captures a small proportion of the match and does not provide any information about the intensities in the remaining time or how these intensities change across the match. To overcome this potential issue, two recent studies examined the distribution of the match-intensity compared with the mean and peak values in male soccer [15], male Australian football and rugby league [33]. In soccer, given the maximal match intensity as 100%, the authors calculated the time and the distance spent at each 10% window for total distance, high-speed running (> 4.2–5.6 m⋅s^−1^), very high speed running (5.6–6.7 m⋅s^−1^), sprint (> 6.7 m⋅s^−1^), acceleration and deceleration (both indicated as variations in speed > 3 m⋅s^2^) distance, and compared them with the mean whole-match values [15]. The authors reported that ~ 61% of total distance, ~ 68% of high-speed running, ~ 80% of very high-speed running, ~ 95% of sprint, ~ 75% of acceleration and ~ 64% of deceleration distance were covered at an intensity greater than the mean [15]. In Australian football and rugby league, 13% and 17%, respectively, of the total distance was performed over 70% of the MIP, and the most distance was covered at ~ 60% of the MIP [33]. These two attempts to describe the distribution of the match intensity with respect to the MIP provide a deeper and more complete analysis of the locomotor activities. However, further studies are required to examine different team sports.

When interpreting the MIP, one must note how contextual factors may influence the values. For example, being a starter or entering as a substitute, the number of minutes played, the time MIP occurred in the match half and the two halves may all concur to vary the total distance determined using the MIP [21]. However, the authors considered the 3-min epoch as the MIP, so the maximal values calculated using shorter durations (e.g. 1-min epoch) may have other characteristics. Indeed, another recent study examined how the team formation, ball-in-play and ball possession impact the 1-min MIP [15]. These factors were also shown to affect the high-speed (> 5.5 m⋅s^−1^) and sprint (> 7.0 m⋅s^−1^) distance values, albeit to a lesser extent [21]. Very similarly, between-quarter decrements in MIP were observed in semi-professional basketball players [34], so that fatigue possibly affects the extent of the MIP [35]. The findings showed that team formation poorly affects the MIP for total distance, high-speed running (4.2 to 5.6 m⋅s^−1^), very high-speed running (5.6 to 6.7 m⋅s^−1^) and sprinting (> 6.7 m⋅s^−1^), while differences were observed for ball-in-play and ball possession [23]. Additionally, positional differences in the MIP were found for total distance, high-speed running (> 4.7 m⋅s^−1^) and sprinting (> 6.1 m⋅s^−1^) in Gaelic football [7], for total distance and high-speed running but not in sprinting in hurling (> 6.1 m⋅s^−1^) [18], total distance and high-speed running in Australian football (> 5.5 m⋅s^−1^) [24] and total distance in rugby union [20] and rugby league [19]. Lastly, differences in MIP were also found in field hockey with different levels of competitions [36]. Taking altogether, the MIP may vary when different contextual factors are considered and should not be taken as unique.

A further possible criticism of the MIP is its match-to-match variability. Indeed, a recent study reported that the coefficient of variation (CV) for the 3-min MIP ranged from 4.6 to 8.2% for total distance, 15.6 to 37.8% for high-speed running (> 5.5 m⋅s^−1^) and 21.1 to 76.4% for sprinting (> 7.0 m⋅s^−1^) [21]. Furthermore, another study in soccer showed that the 1-min MIP CV ranged from 2 to 21% for total distance, 5 to 20% for high-speed running (4.2–5.6 m⋅s^−1^), 5 to 22% for very high speed running (5.6–6.7 m⋅s^−1^), 6 to 23% for sprinting (> 6.7 m⋅s^−1^) and 7 to 24% for acceleration and deceleration (> 3 m⋅s^2^) [23]. Interestingly, the authors of this latter study also calculated the CV for the same metrics using the mean whole-match values [23]. The results appear somewhat surprising: while the CV for total distance was 5–7%, CV for high-speed running was 11–14%, 17–23% for very high-speed running, 25–35% for sprint and 13–17% for acceleration and deceleration, i.e. not different from the CV of the mean with the exception of total distance [23]. These outcomes also reflect the match-to-match variability in mean whole-match values observed in rugby union (between-match CV for high-speed running [4.2–5.5 m⋅s^−1^] = 20 to 28% and very high speed and sprint running [5.6–10 m⋅s^−1^] = 34 to 68%) [37] and league (average speed CV = 6.2% and high-speed (> 5.5 m⋅s^−1^) = 14.4%) [38]. Collectively, this suggests that the variability calculated for the MIP and mean values appears similar. Therefore, while being aware of a certain match-to-match variability in the MIP, it should be still acknowledged that such a variability is likely intrinsic to any match-to-match analysis even when mean values are evaluated, especially for the high-intensity metrics. Consequently, consideration of variability is applicable to all methods of time–motion analysis when determining meaningful changes over time and is not limited to the MIP. As such, practitioners should look to establish the typical variability in any time–motion analysis method to identify meaningful changes in an individual player output during match play using previously established methods [39, 40].

### MIP in the Training Process

A key responsibility for the multidisciplinary team is to plan the training programme. This can be broken down hierarchically from training weeks to sessions to modes (i.e. ‘drills’). The aim is to manipulate and individualise the frequency, intensity and duration of this training hierarchy to optimise the psycho-physiological and biomechanical responses that in turn, maximise the training-induced adaptations of players [41]. However, while duration and frequency can be easily measured, the varied physiological determinants of team sport performance (e.g. VO_2max_, repeated sprint ability, maximal speed and acceleration) provide a challenge to understand the intensity of training prescription across the programme [42]. This is because multiple modes of training are prescribed concurrently including technical–tactical (e.g. SSGs), high-intensity interval training, sprint and repeated sprint training [43–45]. As such, the MIP approach is used to identify the peak intensity of activity for a given duration during competition and then proposed as a benchmark to determine the training intensity of drills [46]. However, since the structure of training modalities (e.g. interval training vs. repeated sprint training) is vastly different, the MIP should not be intended as a universal benchmark for every training mode. In this regard, no previous recommendations have been provided of when the MIP could and should not be reasonably used within practice to target the training intensity. Therefore, the following sections aim to clarify the strengths and weaknesses of the MIP for practitioners as an appropriate intensity benchmark across common team sports training modes.

### When MIP Data Could be Used

For large parts of the calendar year, technical–tactical training modes comprise a large proportion of team sport-specific training. For example, over eight seasons in professional soccer, position-specific-, possession-, SSGs-, tactical- and technical-based training modes accounted for 90% of total training drill prescription across 65,825 drills prescribed by nine head coaches [45]. Conditioning-based training comprised the remaining 10%. As a result, the training prescription across the programme is largely comprised of sport-specific activities whereby the strength and conditioning coach must work with the technical–tactical coaches to blend physical and technical–tactical aims, often referred to as tactical periodisation [47, 48]. In such contexts, some drills will naturally possess greater technical–tactical emphasis, lowering the physical demands, and could be considered a continuum. Given this complexity, understanding the appropriateness of MIP data to evaluate technical–tactical training, including game-based training (SSGs) is an important consideration. It is well established that task constraints (e.g. rules of the game, field dimensions) influence the external intensity of SSGs and other technical–tactical drills [49, 50]. However, the difference between common SSGs prescription and the MIP reported during matches across team sports is unclear.

To the authors’ knowledge, Lacome et al. [47] is the only study to directly compare the peak MIP of different SSGs formats (4v4, 6v6, 8v8, 10v10 [all with goalkeepers]) with official matches (i.e. French Ligue 1 players) across any team sport. In this study, the 1-to-5 min MIP for average-speed and high-speed running (> 4.0 m⋅s^−1^) was lower during the majority of the SSGs (4v4, 6v6, 8v8) to a large extent compared to the MIP of matches for all positions. On the other hand, the MIP for mechanical load (AU), defined as an overall measure of velocity changes calculated using > 2 m∙s^−2^ accelerations, decelerations and changes of direction events, was likely higher during 4v4 SSGs than matches [47]. The findings of the study may suggest that generally these SSGs prescriptions underload in comparison with the MIP of matches and that the manipulation of task constraints possibly influences the external intensity of technical–tactical training such as SSGs.

Investigating the external intensity of a range of SSGs formats (4 min, 5v5 to 10v10) in Premier League soccer, average speeds of 100.5 to 116.5 m·min^−1^ were reported across SSG formats, with the high-speed (> 5.5 to 7 m⋅s^−1^) relative distance between 0.25 and 4 m·min^−1^ [51]. The average speeds reported during these SSGs variants appear to be 23 to 50% lower [51] than the 3 min MIP range reported by position in Premier League football (146 to 167 m·min^−1^) during official matches [21]. Similarly, using identical speed thresholds, high-speed relative distances (5.5 to 7 m⋅s^−1^) reported during the 3 min MIP of Premier League matches are also much greater (6.1 to 9.9 m⋅s^−1^) by 139% and 197% compared to the SSGs variants previously reported [51]. These differences also far exceed the typical match-to-match variability of the 3 min MIP for average-speed (6.5 to 6.9%) and high-speed running (> 5.5 m⋅s^−1^) (21 to 30%) reported during Premier League matches [21]. In other team sports, the average-speed and high-speed (5.0 to 7.0 m⋅s^−1^) intensities of 8v8 ‘onside’ (pass backwards) and ‘offside’ (pass in any direction) games played over 8 min (40 × 40 m playing area) were investigated in professional rugby league players [52]. They reported average-speed and high-speed running (5.0 to 7.0 m⋅s^−1^) for ‘onside’ games to be 101 m·min^−1^ (average speed) and 4.9 m·min^−1^ (high speed) and ‘offside’ games to be 127 m·min^−1^ and 9.5 m·min^−1^. Additionally, the MIP for 5 min was 119–121 m·min^−1^ for average-speed and 13.8 to 19.6 m·min^−1^ for high-speed running (> 5.5 m⋅s^−1^) during international rugby league matches [53]. Therefore, it would appear that only the ‘offside’ game was able to replicate or exceed the average-speed MIP of rugby league match play, although high-speed running was underloaded across both SSG formats.

Collectively, while direct comparisons and firm inferences are difficult due to some method differences (e.g. different microtechnology devices), the findings provide some reasonable suggestions that SSGs training prescription at the elite levels of soccer (e.g. Ligue 1 and Premier League) and rugby league (i.e. National Rugby League) generally appear to be lower than the MIP of matches even when considering their typical variability. The collective findings therefore suggest some rationale that deriving the MIP during matches has the potential to help practitioners to evaluate and improve their technical–tactical training intensities (when appropriate across the training programme) during training modes such as SSGs. To support this, across the training programme, technical–tactical training also involves drills that are planned to be completed at lower intensities, with a greater emphasis on technical–tactical development. For example, the average-speed and high-speed relative distance (> 5.5 m⋅s^−1^) of technical training in professional soccer (i.e. group drills focussing on a skill such as passing, shooting, defending) was 56.8 and 2.1 m·min^−1^, respectively [45]. Conversely, in rugby league, the average-speed and high-speed (> 5.5 m⋅s^−1^) relative distance of skills training was 57 and 2 m·min^−1^, respectively [43]. In female Australian football, the time spent at 60–100% of the MIP for average speed and impulse was at least moderately lower during skill drill training than during match play [6]. Therefore, given that: i) the in-season period comprises the vast majority of a team sport athlete’s calendar year and ii) that technical–tactical coaches possess the primary responsibility for the design of the task constraints of technical–tactical training, MIP data can also be used to evaluate and plan the external intensity of modes where there is a greater technical–tactical emphasis and less physiological development focus.

Arising from this reviewing and planning process is the question of which duration is the most appropriate to choose to evaluate training drills. Establishing an arbitrary upper limit of 10-min length (greater length may be representative of a quarter-match in some team sports and thus outside of the idea of short match intervals), decreasing the interval length overall results in higher MIP values compared to the mean values [18, 27, 54]. Hence, should the primary aim be the determination of the highest peak values, the epochs should be as short as possible. Interestingly, a recent article explored the use of the 4-min epoch in soccer to determine the area per player needed to replicate the official match MIP during different formats of SSGs [22]. The rationale for having chosen the 4-min epoch was that the SSGs mean duration deriving from the elite soccer practice was 4 min [22], possibly defined as the ‘optimal’ duration in this context [55]. Notwithstanding, due to the variability of the training drills and their durations prescribed across a training period and the variability of the activities completed by players within each drill, it would be difficult to establish the optimal interaction between the intensity and duration required for optimising the training-induced adaptations. Pragmatically, it would be logical for practitioners to compare each drill to the MIP during match play for the exact duration of the drill. For example, average speed during a 4-min 30-s drill completed by a player can be compared to those players with a 4 min 30 s average-speed MIP during competition. Using the same idea, shorter epochs may be used for position-specific drills depending on how each peak load is distributed in the matches [15], so as to eventually implement the training load with further drills to stimulate the players properly. While research typically reports arbitrarily a 1 to 10 min duration in 1 min increments, smaller increments can be used (e.g. 30 s increments), and the MIP for any time duration can be estimated using the power-law relationship described earlier. On these bases, we suggest that the choice of the MIP duration should depend on the purposes of the analysis and may be reviewed and targeted accordingly.

Despite these comparisons, it must also be balanced that there is a conceptual assumption that training at these external intensities during the team ‘sport-specific’ activities (either during official matches or SSGs) is actually of an appropriate intensity to stimulate training-induced adaptations and the development of the physiological determinants of performance (e.g. speed development, aerobic capacity and repeat sprint ability) [21]. However, there is also some evidence that SSGs prescription over a training programme can improve such determinants [56, 57]. Yet, it must also be acknowledged that there are methodological limitations to some of these studies (e.g. no information documenting the training intervention itself, some absence of control groups) [57]. At most, it is inconclusive whether MIP data can or cannot be a useful method to evaluate the training intensities of technical–tactical focussed training and further research is required. Particularly, specific training intervention studies using MIP data within a training programme are required to confirm any assumptions.

### When MIP Data Should not be Used

Firstly, it is important to highlight that MIP data are captured during competition whereby players are completing team sport-specific activities, which include changes of speed and direction, and in some sports tackling and grappling are therefore unlikely to be moving in a constant linear direction. Therefore, the context of the data is important when considering its applicability to training modes across the training process. For example, as described earlier, the peak average speed across a 1-min duration for rugby league, rugby union, and Australian Rules football was reported to be 165 (2.75 m·s^−1^), 171 (2.85 m·s^−1^) and 210 (3.5 m·s^−1^) m·min^−1^, respectively [20, 24, 28–31]. The average speed during maximal time trial tests, which are reflective of minimal speed needed for the attainment of VO_2max_, has also been examined across studies within these sports [58–60]. The average speeds during a 1.2-km time trial in professional rugby union players was reported to be 4.2 and 4.9 m⋅s^−1^ for forwards and backs, respectively (31). Similarly, studies investigating the 2 km time trial performance in professional rugby union and sub elite Australian Rules footballers reported average speeds of 3.8 and 4.6 m⋅s^−1^, respectively (3, 30). By considering the difference in average speed between the MIP during match play and time trial performances, it is clear and obvious that MIP data are much lower than a player’s estimated speed at VO_2max_ [58–60]_._ Therefore, such data provide no benefit in the prescription or evaluation of intensity for training modes such as interval-based training. This is because maximising the time spent at VO_2max_ is one key-aim of the interval training prescription, and the peak average speeds during competition are therefore much lower [61, 62]. Consequently, by using MIP as a target for this type of training, adaptations are unlikely to occur. As a result, we recommend that when evaluating and prescribing the intensity of running-based training, practitioners do not use MIP values during competition. Instead, draw upon well-established methods of individualising training intensity during this training mode, such as percentage of termination speed of incremental or critical speed tests, maximal aerobic speed from a time trial or direct physiological markers such as speeds corresponding to VO_2max_ or blood lactate threshold landmarks [63, 64]. Similarly, if the speeds derived from the MIP are lower than that achieved at VO_2max_, using MIP data to inform the prescription of linear speed and repeated sprint training modalities would also clearly be inappropriate as these intensities are designed to be close to the maximal speed and acceleration intensity capable by the athletes. To summarise, MIP data, albeit ‘maximal’ in the match context, do not reflect the players’ maximal aerobic capacity or their maximal speed, and should not be used as its substitute when implementing running-based training for this purpose. This may indeed lead to underloading the players, should MIP be considered as the benchmark.

### Limitations and Future Directions of the MIP

Conceptually, the method of determining MIP has focussed on its application to measurements of external intensity. Indeed, the MIP is simply a method of analysing the whole time series signal of a measurement and can be applied to any variable that measures over a granular period. The aim of the training programme is to prescribe activities that maximise the physiological responses to drive adaptations of the determinants of performance. Therefore, direct measurements of psycho-physiological response to derive training intensity targets for training drills  should be integrated. This could be achieved currently via the use of the session ratings of perceived exertion (sRPE), differential ratings of perceived exertion (dRPE) or heart rate.

While sRPE and dRPE methods are very useful to provide an evaluation of the overall session intensity and can be used to evaluate the average intensity of individual training drills [43, 44], they can only provide an average measure of intensity of the session, and so therefore, a moving average cannot be applied to identify the maximal intensity for a given duration. This is because only one value can be produced from these methods. The use of heart rate to identify a maximal moving average for a given duration is promising, as a continuous time series signal can be extracted for the full drill so to integrate measures of internal intensity to the match locomotor activities. Further future developments in wearable technology capable of measuring a wider range of physiological responses (e.g. skin and sweat sensors, wearable garments) could help to ‘bridge the gap’ between the concept of internal intensity and its measurement across training modes within a team sport training programme [65]. Consequently, researchers and practitioners should remain cognisant of the strengths and limitations of all measurements used to evaluate different training modalities across the complexity of the overall training programme. Finally, irrespective of whether the measurement reflects the external or internal intensity, it is common for research studies to investigate the MIP for different measurements in isolation, and not concurrently, which is likely to occur during match play. For example, the MIP for average speed could have been achieved alongside the peak acceleration MIP. Future work should look to address this interaction and establish the typical timings of the MIP between measurements.

## Conclusions

Identifying the maximal moving average for a given duration of time appears to produce substantially greater values than when averaged across the whole match in team sports. The magnitude of such differences appears to be greater than the magnitude of the MIP match-to-match variability or the effects of contextual factors on the MIP. Since team sport training programmes are complex and comprise a multitude of training modes, we have rationalised that MIP data could be useful to inform the intensity of technical–tactical training modes such as SSGs, which comprise a large proportion of team sport training programmes. Notwithstanding, MIP data are inappropriate to prescribe the intensity of running-based activities such as interval, repeated sprint and linear speed training modes. Future research should investigate the effects of an MIP-focussed training intervention on the physiological determinants of performance. As such, practitioners should consider the strengths and the weaknesses of the MIP data when designing training routines.

## Data Availability

Not applicable.
